# A *SLC14A1* c.230G>A variant causes loss of Kidd antigen expression

**DOI:** 10.3389/fimmu.2026.1922509

**Published:** 2026-10-29

**Authors:** Xiaozhen Hong, Jingjing Zhang, Yanling Ying, Xianguo Xu, Faming Zhu

**Affiliations:** Blood Center of Zhejiang Province, Hangzhou, China

**Keywords:** antigen expression, function analysis, Jk(a-b-) phenotype, single molecule sequencing, SLC14A1

## Abstract

**Background:**

The Kidd (Jk) blood group system is defined by the Jk^a^, Jk^b^ and Jk3 antigens encoded by the *SLC14A1* gene. The Jk(a-b-) phenotype, characterized by the absence of these antigens, is a rare blood group phenotype with a low population frequency, and its molecular basis is primarily associated with variants in the *SLC14A1* gene. Herein, we identified an individual with the Jk(a-b-) phenotype carrying a variant, and evaluated whether this variant affects Jk antigen expression.

**Methods:**

The Jk(a-b-) phenotype was identified by conventional serological typing. The coding sequence of the *SLC14A1* gene was analyzed using polymerase chain reaction sequence-based typing (PCR-SBT), and haplotype analysis was performed using single-molecule sequencing. The functional impact of the variant was evaluated through *in vitro* transfection and expression assays, and Jk^b^ antigen expression on transfected cells was assessed by flow cytometry. The potential functional impact of the variant was also evaluated using *in silico* prediction tools.

**Results:**

Jk^a^, Jk^b^ and Jk3 antigens were absent in the proband. PCR-SBT identified three heterozygous single-nucleotide variants (SNVs) and two homozygous SNVs across four exons of the *SLC14A1* gene. Single-molecule sequencing revealed two haplotypes: one comprising c.342-1G>A, c.588A>G, and c.838G>A, and the other comprising c.230G>A, c.499A>G, c.588A>G, and c.838G>A. *In silico* analysis predicted c.230G>A to be a deleterious variant and indicated that it altered interactions with neighboring atoms. Functional assays showed that cells transfected with JK(c.230G>A+c.499A>G+c.588A>G+c.838G>A) did not express the Jk^b^ antigen, while cells transfected with the JK*02 allele or JK(c.499A>G+c.588A>G+c.838G>A) exhibited high levels of Jk^b^ antigen expression.

**Conclusions:**

A JK null allele and its critical SNV were identified, and the functional effect of the variant was confirmed through transfection and expression studies.

## 1 Introduction

The Kidd blood group system is one of the recognized human blood group systems and comprises three antigens: Jk^a^ (009001), Jk^b^ (009002), and the high-frequency antigen Jk3 (009003) (1). The Kidd glycoprotein is encoded by the *SLC14A1 (JK)* gene located on chromosome 18 (2, 3). This glycoprotein contains 10 putative transmembrane domains, with both the N- and C-termini located intracellularly. The *SLC14A1* gene spans approximately 30 kb and consists of 10 exons. Exons 1 and 2, together with part of exon 3, form the untranslated region, while exons 3 to 10 encode the mature protein (4). The Jk^a^/Jk^b^ polymorphism results from the c.838G>A substitution in exon 8 of the *SLC14A1* gene, which leads to an Asp280Asn amino acid change in the fourth extracellular loop of the Kidd glycoprotein (5). The corresponding alleles are designated JK*01 (JK*A) and JK*02 (JK*B).

Based on the expression of Jk^a^ and Jk^b^ antigens on red blood cells, four phenotypes are recognized: Jk(a+b-), Jk(a-b+), Jk(a+b+), and Jk(a-b-). The Jk(a+b-), Jk(a-b+), and Jk(a+b+) phenotypes occur at varying frequencies in different populations. In contrast, the Jk(a-b-) phenotype is rare, which was first described in 1959 (6) and is characterized by the absence of Jk^a^, Jk^b^, and the high-frequency antigen Jk3 on red blood cells. Previous studies have reported that the frequency of the Jk(a-b-) phenotype is 0.002% (14/638,460) among Japanese individuals (7) and 0.008% (16/201,194) among Chinese individuals (8). However, this phenotype occurs at a relatively higher frequency among Polynesians, with a reported prevalence of 0.27% (47/17,300) (9).

Due to the role of the Kidd glycoprotein as a urea transporter (10, 11), red blood cells with the Jk(a+b-), Jk(a-b+), and Jk(a+b+) phenotypes undergo lysis within 1 minute in 2 mol/L urea, whereas red blood cells with the Jk(a-b-) phenotype require at least 30 minutes to lyse (12). Therefore, the urea lysis test can be used as a laboratory screening method for the Jk(a-b-) phenotype. Multiple individuals with the Jk(a-b-) phenotype have now been identified (13–15). Individuals with Jk(a-b-) phenotype may produce anti-Jk3 antibody through blood transfusion and pregnancy pathways. Anti-Jk3 antibody has been responsible for severe acute and delayed hemolytic transfusion reactions (16, 17). Therefore, it is necessary to select the blood from Jk(a-b-) phenotype individuals for transfusion if anti-Jk3 antibody was existed. Due to the low proportion of Jk(a-b-) phenotype in the populations (7, 8), multiple blood services have regularly screened the Jk(a-b-) phenotype in blood donors in order to meet potential clinical transfusion needs (8, 18). However, the majority of Jk(a-b-) individuals from blood donors did not produce anti-Jk3 antibodies. The molecular basis of the Jk(a-b-) phenotype is associated with variants in the *SLC14A1* gene (1, 4, 19). According to the International Society of Blood Transfusion (ISBT) Blood Group Database (released on 1 May 2026), 61 JK null alleles associated with the Jk(a-b-) phenotype have been registered (https://blooddatabase.isbtweb.org). Most JK null alleles contain a single nucleotide variant on either the JK*01 or JK*02 background, whereas some null alleles harbor two or more nucleotide variants.

In the present study, we identified a voluntary blood donor with the Jk(a-b-) phenotype who harbors a c.230G>A variant in the *SLC14A1* gene. The variant was characterized using polymerase chain reaction sequence-based typing (PCR-SBT) and single-molecule sequencing technology. In addition, *in vitro* transfection and expression studies were performed to determine whether the c.230G>A variant affects Jk antigen expression.

## 2 Materials and methods

### 2.1 Samples

The specimens from screening individuals including the proband were obtained from blood donors who participated in voluntary blood donation at the Blood Center of Zhejiang Province, China. Additional specimens were collected from the proband’s parents. All specimens were collected after obtaining informed consent.

### 2.2 Screening for the Jk(a-b-) phenotype

The specimens were screened for the Jk(a-b-) phenotype using a 2 mol/L urea lysis test according to our previous report (18). Briefly, 20 µL of a 2%-5% (v/v) red blood cell (RBC) suspension and 100 µL of 2 mol/L urea solution were added to microplate wells, mixed thoroughly, and centrifuged at 450×g for 1 min. Hemolysis was then observed. Specimens showing no hemolysis were further tested for Jk^a^ and Jk^b^ antigens using the serological methods.

### 2.3 Jk^a^, Jk^b^ and Jk3 antigens were determined using serological analysis

Specimens with positive screening results in the urea lysis test were further confirmed using anti-Jk^a^ and anti-Jk^b^ IgM monoclonal antibodies according to the manufacturers’ instructions (Sanquin, Amsterdam, Netherlands; Werfen, Shanghai, China), as well as in-house anti-Jk3 antibody [derived from an individual with the Jk(a-b-) phenotype from Shanghai Blood Center, China, which was not agglutinated with RBCs of Jk(a-b-) phenotype, but agglutinated with RBCs from Jk(a+b-), Jk(a+b+) and Jk(a-b+) phenotypes]. Briefly, a 2%–5% (v/v) red blood cell suspension was mixed with anti-Jk^a^ or anti-Jk^b^ in a tube, incubated, centrifuged, and examined for RBC agglutination. In addition, the Jk^a^, Jk^b^ antigen expression in the proband was assessed using an absorption-elution test. For Jk3 antigen detection, the anti-human globulin method using a microcolumn gel card was applied. Three RBCs from Jk(a+b-), Jk(a+b+) and Jk(a-b+) phenotypes respectively in the panel cells (Shanghai Blood Bio-technology, Shanghai, China) were used as controls.

### 2.4 Analysis of the *SLC14A1* gene sequence using PCR-SBT

The sequences of exons 3 to 10 and their flanking regions of the *SLC14A1* gene were analyzed using PCR-SBT as described in our previous report (20). First, genomic DNA was extracted from the specimens using the MagDNA Pure LC DNA Isolation Kit according to the manufacturer’s instructions and our previous protocol (21). Subsequently, exons 3–10 of the *SLC14A1* gene were amplified and sequenced using the Sanger sequencing method. The reference sequence of the *SLC14A1* gene was obtained from GenBank (NG_011775.4), and variants were identified based on nucleotide polymorphisms relative to this reference. The primers and PCR conditions are provided in **Supplementary File 1**.

### 2.5 Amplification of the full-length *SLC14A1* gene

The full-length region of the *SLC14A1* gene, spanning from the 5′UTR to exon 10, was amplified using two amplicons of 19,172 bp and 16,945 bp, respectively, and the sequences of two amplicons overlap a 7305 bp region (**Supplementary Figure 1**). The primer sequences for amplicon 1 were as follows: forward primer Kidd-F1, 5′-TTCATCCCAAAGCAGGCC-3′; reverse primer Kidd-R1, 5′-ACTGCRTGGGACCTAGCT-3′. For amplicon 2, the primers were: forward primer Kidd-F2, 5′-CCAGTTCTCCGAACCTCCTT-3′; reverse primer Kidd-R2, 5′-TGCCTACCTGGCTAAT-3′. The total PCR reaction volume was 20 µL, containing 1×PCR buffer, 0.5 mM dNTPs, 0.2 µM of each forward and reverse primer, 1U KOD FX Neo DNA polymerase (Toyobo, Osaka, Japan), and approximately 50 ng genomic DNA, with nuclease-free water added to a final volume of 20 µL. The PCR cycling conditions were as follows: initial denaturation at 94 °C for 2 min, followed by 33 cycles of 98 °C for 10 s, 68 °C for 15 min, and a final extension at 68 °C for 10 min.

### 2.6 Construction of the library for a single-molecule sequencing

The amplicons were pooled and analyzed using third-generation sequencing based on nanopore technology (Oxford Nanopore Technologies, Oxford, UK). Briefly, the amplicons were first purified using a nucleic acid purification kit (Agencourt^®^ AMPure^®^ XP, Beckman Coulter, Shanghai, China). A sequencing library was then constructed using the Multiple Samples DNA Library Prep Kit for Ligation Sequencing (Baiyitech, Hangzhou, China) for Oxford Nanopore Technologies (ONT, Oxfordshire, UK), including DNA end repair, barcode ligation to index each specimen before pooling, native adaptors ligation and purification, library quantification, preprocessing and sample loading of sequencing chips. The library was sequenced on a MinION platform using an R10 flow cell (ONT). The Phred quality score (Q score) threshold was set at 10 when basecalling of the raw nanopore data was generated to FASTQ files in the MinION platform. FASTQ files were imported into a commercial software (Weihe Company, Jiangsu, China) for haplotype assignment of the *SLC14A1* gene based on the reference sequence (GenBank ID: NG_011775.4). All procedures were performed according to the manufacturer’s instructions.

### 2.7 *In silico* prediction for missense variants

The potential functional impact of the variants was evaluated using bioinformatics tools, including PROVEAN (https://www.jcvi.org/research/provean), MutPred2 (http://mutpred.mutdb.org), FATHMM (http://fathmm.biocompute.org.uk/), MutationTaster (https://www.mutationtaster.org/), and PhD-SNP (http://snps.biofold.org/phd-snp/phd-snp.html), according to the respective developers’ instructions.

### 2.8 Structural modeling

Using the X-ray crystal structure of the SLC14A1 (PDB: 6QD5) obtained from the Research Collaboratory for Structural Bioinformatics Protein Data Bank (RCSB PDB) as a template, initial structural models of the wild-type and variant SLC14A1 proteins were constructed. Structural analysis and visualization were performed using the PyMOL Molecular Graphics System (Schrödinger, LLC, New York, NY, USA; version 3.1.8).

### 2.9 Synthesis of a specific expression plasmid

Three recombinant plasmids were constructed, including a wild-type (Jk^b^, c.838G>A) construct and two variant constructs: JK(c.230G>A+c.499A>G+c.588A>G +c.838G>A) (defined as JK^V1^), and JK(c.499A>G+c.588A>G+c.838G>A) (defined as JK^V2^). The cDNAs encoding the wild-type and variant sequences were synthesized and cloned into the pcDNA3.1(+)-Myc-His A expression vector (Jinsirui Company, Nanjing, China). All recombinant plasmids were validated by Sanger sequencing.

### 2.10 Cell transfection and screening with a specific expression plasmid

Three specific recombinant expression plasmids and the original pcDNA3.1(+) vector were transfected into CHO cells (Cell Bank of the Typical Culture Collection Committee, Chinese Academy of Sciences, Beijing, China) using a commercial liposome-based transfection reagent (Lipo8000, Beyotime, Shanghai, China) according to the manufacturer’s instructions. Stable CHO cell lines were established through G418 selection, as previously reported (22).

### 2.11 Analysis of Jk^b^ antigen expression on the transfected cells

Jk^b^ antigen expression on CHO cells was detected using flow cytometry. Three to five clones of different transfection CHO cells were chosen for analysis which is from one transfection. Briefly, 50 µL of cell suspension (approximately 5×10^5^ cells) was mixed with 50 µL of IgM anti-Jk^b^ antibody (Sanquin, Amsterdam, Netherlands; clone:MS8) and incubated for 1 h. After washing, Alexa Fluor 647 affinipure F(ab’)2 fragment goat anti-human IgG (Jackson ImmunoResearch Laboratories Inc., Lancaster, Pennsylvania, USA; catalog number 109-606-006;APC channel detection) was added and incubated at room temperature for 30 min. The cells were then washed and resuspended in 500 µL phosphate-buffered saline (PBS) prior to analysis on a BD FACSVerse flow cytometer(Becton, Dickinson and Company,Franklin Lake, New Jersey, USA). The gating strategy was according to our previous report (22). At first, intact cells were first identified based on forward scatter (FSC-A) versus side scatter (SSC-A) dot plots to exclude cellular debris. Then, cells stably transfected with the original vector were used as a negative control to establish the threshold for positive fluorescence. Because only a single fluorescent channel was used in this study, fluorescence compensation was not required. A total of 20,000 events within the pre-defined cell gate were acquired for each specimen by BD FACSuite software. Data were analyzed using BD FlowJo v10.8. Jk^b^ antigen expression on the cell surface was determined based on mean fluorescence intensity (MFI).

### 2.12 Statistical analysis

Comparison of MFI values between different transfection cells by Student’s t test (unpaired, two-tailed) using GraphPad Prism 10.0 software (GraphPad Software company, Boston, Massachusetts, USA). p<0.05 was considered statistically significant.

## 3 Results

### 3.1 Jk(a-b-) phenotype was identified by the serology method

A total of 20,000 voluntary blood donor specimens were screened for the Jk(a-b-) phenotype using a urea hemolysis assay, and only one individual was identified as resistant to hemolysis. The RBCs of the proband with anti-Jk^a^, anti-Jk^b^ showed no agglutination using the conventional tube method and absorption-elution test. In the controls, RBCs of Jk(a+b-) and Jk(a+b+) phenotypes with anti-Jk^a^ showed 2+ and 1+ agglutination strength, while RBCs of Jk(a+b+) and Jk(a-b+) phenotypes with anti-Jk^b^ showed 1+ and 2+ agglutination strength. For Jk3 antigen analysis, 2+ agglutination strength were found in the RBCs of Jk(a+b-), Jk(a+b+) and Jk(a-b+) phenotypes with the anti-Jk3 antibody using a microcolumn gel card, but no agglutination was found in the proband. Therefore, No Jk^a^ or Jk^b^ or Jk3 antigens were detected in the proband. No unexpected antibodies were detected in the proband’s plasma. The corresponding data for the proband’s parents are also presented in **Table 1**. The proband’s other blood group phenotypes were type A, ccDEE, and MN.

**Table 1:** RBC phenotypes and nucleotide information of the proband and his parents.

Sample	Anti-Jk^a^	Anti- Jk^b^	Phenotype	Genotyping				
				*SLC14A1*				
				PCR-SBT	Haplotype 1		Haplotype 2	
Proband	0	0	Jk(a-b-)	c.230G/A c.342-1G/A c.499A/G c.588G/G c.838A/A	c.230G>A c.499A>G c.588A>G c.838G>A	Variant allele	c.342-1G>A c.588A>G c.838G>A	JK*02N.29
Father	0	+	Jk(a-b+)	c.342-1G/A c.588G/G C.838A/A	c.342-1G>A c.588A>G c.838G>A	JK*02N.29	c.588A>G c.838G>A	JK*02+c.588G
Mother	+	0	Jk(a+b-)	c.230G/A c.499A/G c.588A/G c.838G/A	c.838G	JK*01	c.230G>A c.499A>G c.588A>G c.838G>A	Variant allele

### 3.2 The sequence of the *SLC14A1* using Sanger sequencing

Three heterozygous single-nucleotide variants (SNVs) and two homozygous SNVs were identified across four exons in the proband (**Figure 1**; **Table 1**), including c.230G>A (rs2511219464, p.Ser77Asn) in exon 4, c.342-1G>A at the splice site, c.499A>G (rs2298719, p.Met167Val) and c.588A>G (rs2298718, p.Pro196Pro) in exon 6, and c.838G>A (rs1058396, p.Asp280Asn) in exon 8. In addition, three and four SNVs were identified in the proband’s father and mother, respectively (**Table 1**).

**Figure 1:**
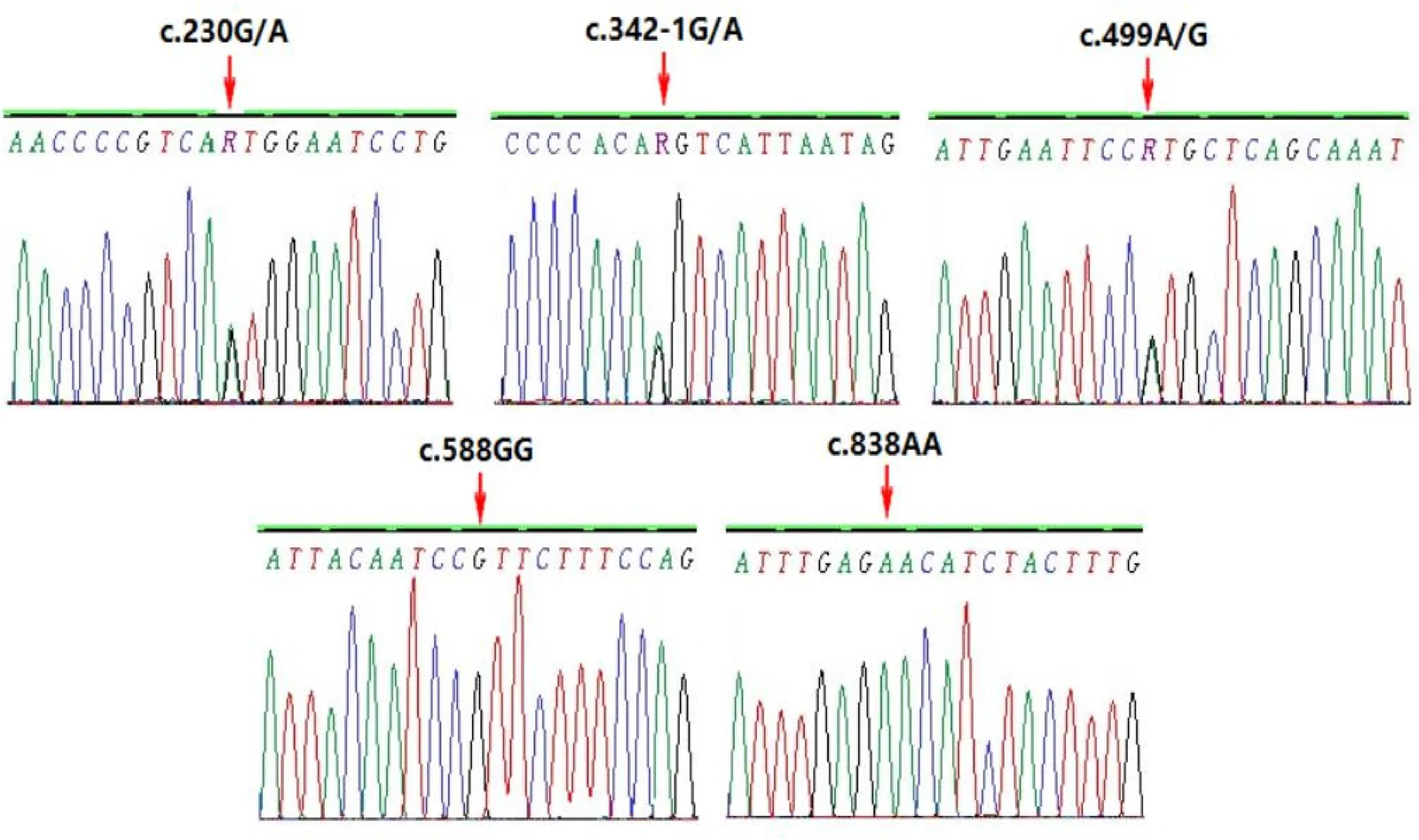
The SNVs of the *SLC14A1* gene in the Jk(a-b-) donor by Sanger sequencing: c.230G>A, c.342-1G>A, c.499A>G,c.588GG,c.838AA. The position of the arrow is indicated the different variants.

### 3.3 Identification the haplotype using the single-molecule sequencing method

In the single-molecule sequencing, it was obtained 53,368 and 71,683 reads for different two amplicons, and average coverage are 345 and 506. A haplotype comprising c.342-1G>A, c.588A>G, and c.838G>A within the *SLC14A1* gene in the proband was identified using single-molecule sequencing and designated as JK*02N.29 (**Table 1**). The second haplotype consisted of c.230G>A, c.499A>G, c.588A>G, and c.838G>A and was designated as a JK*02 allele (c.230G>A variant allele), and it has been officially nominated as JK*02N.35 by ISBT Working Party for Red Cell Immunogenetics and Blood Group Terminology. The proband’s father carried the JK*02N.29 and JK*02 (with c.588G) alleles, whereas the proband’s mother carried the JK*01 and c.230G>A variant allele. The sequence of the allele with c.230G>A has been submitted to GenBank (accession PX780294). The c.230G>A variant is present in the SNP database (rs2511219464), although its allele frequency is not reported.

### 3.4 *In silico* prediction for missense variants

Three missense variants, including c.230G>A, c.499A>G and c.838G>A were predicted to affect the function of the *SLC14A1* gene by multiple *in silico* tools (**Table 2**). However, the predicted impact of these variants varied depending on the algorithm used. c.230G>A variant was classified as deleterious or disease-associated using the PROVEAN, SIFT, MutPred2 and PhD-SNP tools, but as tolerated by FATHMM and MutationTaster tools. c.499A>G and c.838G>A variant were predicted as tolerated or benign by all tools.

**Table 2:** The effect of missense variants in silico prediction using different tools*.

SNV	Amino acid change	PROVEAN	SIFT	MutPred2	FATHMM	MutationTaster	PhD-SNP
		Score	Score	Score	Score	Score	Neutral/disease
c.230G>A	p.Ser77Asn	-2.76	0.01	0.69^#^	0.45(Tolerated)	46 (Tolerated)	Disease
c.499A>G	p.Met167Val	1.21	1.00	0.04	0.98(Tolerated)	21 (Benign)	Neutral
c.838G>A	p.Asp280Asn	-0.22	0.59	0.13	0.74(Tolerated)	23 (Benign)	Neutral

*PROVEAN prediction (cutoff = -2.5; deleterious or neutral), SIFT (cutoff = 0.05; tolerated or damaging); MutPred2 (score range 0-1), FATHMM (deleterious or tolerated), MutationTaster (deleterious or benign), PhD-SNP (disease or neutral). ^#^Alter transmembrane protein.

### 3.5 Structural modeling

The predicted structures of the wild-type and variant forms are shown in **Figure 2**. In the wild type, Ser77 formed polar interactions with Asn74, Ile81, and Ala248 (**Figure 2a**), including two hydrogen bonds (3.2 Å and 3.0 Å) with Asn74, one hydrogen bond (2.9 Å) with Ile81, and one hydrogen bond (3.1 Å) with Ala248. In the p.Ser77Asn variant, these polar interactions were altered, with Asn77 forming polar interactions with Asn74 and Ala248 (**Figure 2b**), including two hydrogen bonds (3.2 Å and 3.0 Å) with Asn74 and one hydrogen bond (2.8 Å) with Ala248; however, the hydrogen bond with Ile81 was lost.

**Figure 2:**
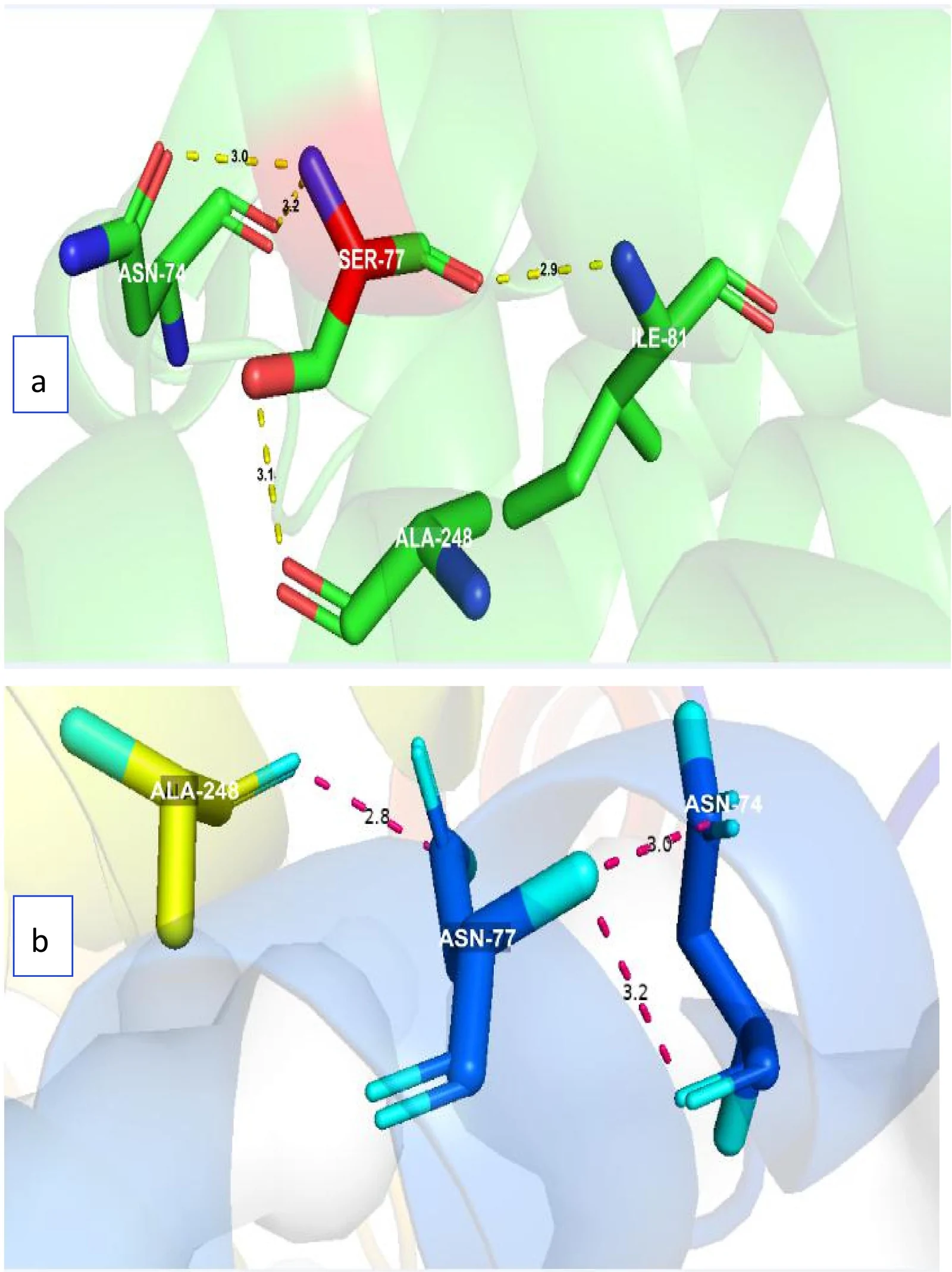
The interaction between wild-type and variant proteins in the spatial structure of other amino acids. **(a)** Shows wild type, **(b)** shows variant type. The dashed line in the figure 2 represents the connection of hydrogen bonds, and the number represents the distance.

### 3.6 Analysis of Jk^b^ antigen expression on the transfected cells

Cells transfected with JK*02 (Jk^b^) and JK^V2^ exhibited high-level expression of the Jk^b^ antigen (**Supplementary Figure 2**). In contrast, cells transfected with JK^V1^ showed no detectable Jk^b^ antigen expression, with MFI values comparable to those of the vector control group (negative control). The MFI values of difference transfection CHO cells are listed in the **Table 3**. The MFI values in the CHO with JK^b^ vector or CHO with JK^V2^ vector are significantly higher than that of the CHO with original vector (p<0.05). However, there is no difference in MFI values between the CHO with JK^V1^ vector and CHO with original vectort (p>0.05).

**Table 3:** Quantitative MFI data from CHO cells with difference transfection.

Cells	n	MFI values	SD
CHO with original vector	3	1786	62
CHO with JK^b^ vector	4	7596	930
CHO with JK^V1^ vector	3	2012	818
CHO with JK^V2^ vector	5	5845	728

CHO with JK^b^ vector vs CHO with original vector, t=8.87, p<0.01. CHO with JK^V1^ vector vs CHO with original vector, t=1.47, p>0.05.

CHO with JK^v2^ vector vs CHO with original vector, t=7.21, p<0.01. CHO with JK^V1^ vector vs CHO with JK^b^ vector, t=8.24, p<0.01

CHO with JK^V2^ vector vs CHO with JK^b^ vector, t=3.18, p<0.05. CHO with JK^V1^ vector vs CHO with JK^v2^ vector, t=6.91, P<0.01.

## 4 Discussion

The Jk(a-b-) phenotype is rare in the general population and is commonly screened in blood donors using urea hemolysis tests in many laboratories in China (8, 18). Although one case of the Jk(a-b-) phenotype was identified from 20,000 specimens in the present study and additional cases have been reported previously (23) in our laboratory, these findings suggest that its frequency of the Jk(a-b-) phenotype is very low in the Chinese population. In addition, it should be noted that heterozygous nonfunctional alleles of the *SLC14A1* gene may be not identified by the urea test.

The c.838G>A variant in the *SLC14A1* gene is responsible for the Jk^a^/Jk^b^ polymorphism (5). Moreover, several other polymorphic sites exist in this gene, including c.499A>G and c.588A>G. c.588A>G is not associated with Jk^a^ or Jk^b^ antigen expression (4, 19). However, whether c.499A>G alone or with c.838G>A affects the antigen expression has not been reported, but they are not associated with the Jk(a-b-) phenotype according to the ISBT Blood Group Database (https://blooddatabase.isbtweb.org.). The Jk(a-b-) phenotype is primarily caused by variants in the *SLC14A1* gene and is associated with 61 null alleles recorded in the ISBT Blood Group Database, all of which involve nucleotide changes in the *SLC14A1* gene. These variants may result in single amino acid substitutions, premature protein truncation, or exon skipping, ultimately leading to the absence of Jk antigen expression on red blood cells.

A total of five nucleotide changes were identified in the proband; however, c.499A/G, c.588G/G, and c.838A/A have previously been reported as not associated with the Jk(a-b-) phenotype (1, 4, 19). Therefore, further analysis focused on c.230G/A and c.342-1G/A. Currently, third-generation long-read sequencing (TGS) has been used for resolving genotype-phenotype discrepancies in the Kidd system (24). In additional, TGS has also applied for haplotype assignment of the *SLC14A1* null and weak-expression alleles (13, 24, 25). In this study, single-molecule sequencing showed that c.230G>A and c.342-1G>A were located on different haplotypes. The c.342-1G>A variant in haplotype 2 is a characteristic change of the JK*02N.29 allele, which can lead to exon 5 skipping during transcription and has previously been associated with the Jk(a-b-) phenotype (26, 27). c.230G>A variation has previous found in the Jk(a-b-) phenotype from Shangdong, China (28). The infer haplotype is c.230G>A+ c.499A>G+c.588A>G+c.838G>A (described as JK-02-230A allele in the previous study), which is same as that of our report. However, the functional characterization of the variation was not analyzed in the previous study (28). Therefore, the potential functional role of c.230G>A was further investigated. First, *in silico* predictions classified the variant as deleterious, and structural analysis indicated that it altered hydrogen-bond interactions with neighboring residues, suggesting a potential impact on protein structure and function. Furthermore, no Jk antigen expression was detected on the cell membrane of cells transfected with the c.230G>A recombinant construct *in vitro*. These results indicate that c.230G>A may be abolished antigen expression. According to the ISBT Blood Group Database (https://blooddatabase.isbtweb.org), several exon 4 variants, including c.202C>T, c.220A>G, c.222C>A, and c.299C>T, are associated with the Jk(a-b-) phenotype, whereas c.226G>A and c.277G>A are associated with weak Jk antigen expression. Further investigation these variants using protein spatial structural analysis, which will be benefit to understand the mechanism for antigen lack or weak expression of these variants in exon 4.

However, some limitations were existed in this study. Firstly, a reduction in antigen staining could result from reduced total protein expression, impaired folding, impaired trafficking to the plasma membrane, or alteration of the antigenic epitope. However, these experiments did not preformed. Although the flow-cytometry experiment in our study demonstrates loss of detectable Jk^b^ antigen on the transfected cells, it cannot fully establish the mechanism. Secondly, CHO cells were selected for functional analysis for the *SLC14A1* gene in this study as previous reports (13, 29). However, endogenous SLC14A1 expression in the CHO cells may be affected the results. Thirdly, the function of loss of one predicted hydrogen bond in structural analysis was not further evaluated using the experiments. Therefore, the results of the structural analysis cannot be used as mechanistic evidence. Finally, the proband carries c.342-1G>A and c.230G>A on different haplotypes. The c.342-1G>A variant is already a well-established null allele (26, 27), but it was not established effect of c.342-1G>A from the experimental evidence in this study.

## 5 Conclusions

In conclusion, an individual with the Jk(a-b-) phenotype was identified by serological analysis, in whom the c.230G>A and c.342-1G>A variants located on different haplotypes by long-read sequencing. c.230G>A variant can be significantly decreased the MFI value of transfection CHO cells with corresponding antibodies in the functional experiment. In additional, hydrogen-bond interactions around Ser77/Asn77 was changed based on PDB structure 6QD5 by structural analysis and c.230G>A variant may be as deleterious or disease-associated using *in silico* tools. However, these data was not confirmed by independent functional experiments.

## Data Availability

The datasets presented in this study can be found in online repositories. The names of the repository/repositories and accession number(s) can be found in the article/**Supplementary Material**.
