# Supplementary material for: A SLC14A1 c.230G>A variant causes loss of Kidd antigen expression
Source: Front Immunol. 2026 Oct 29;17:1922509. doi: 10.3389/fimmu.2026.1922509 (PMC13754721; doi:10.3389/fimmu.2026.1922509)
Supplement: Supplementary file 1 [file Table1.docx]

Supplementary Material

# 1 Supplementary Data

**The PCR-SBT primers and amplified condition for the *SLC14A1 gene***

**Supplemental Table 1 The amplification and sequencing primers for 3 to 10 exon of the *SLC14A1* gene**

| Primer | Sequence 5’-3’ |
| --- | --- |
| JK-E3F1 | GGA AAA TGG TGC TCT CTT AG |
| JK-E3R1 | TAA AAG TAG AAA AAT GGT GAG TAA |
| JK-E4F1 | GCC TGT GGT TGA AGA GTA TC |
| JK-E4R1 | ATT CCC TGA CCT CTG ACT AA |
| JK-E5F1 | GCT CAC CCA GGA AAT GTC |
| JK-E5R1 | AAC TCC CAT TCC CTC CTT |
| JK-E6F1 | AAA CAA AGC CCC TCC AGA GT |
| JK-E6R1 | GCC ACC ATA GTC CTG AGC AT |
| JK-E78F1 | AGG AGT TTG TGG GTG TCC TG |
| JK-E78R1 | GGG ACG TCA CTT GTC CTG TT |
| JK-E9F1 | GCT CAT GCT TGT AAT CAG G |
| JK-E9F1 | AGT GGA CTT CAG GAG CAT T |
| JK-E10F1 | CAG AAT CCA GAG CAA TGC CC |
| JK-E10R1 | TTG CTG AGA CCC TGG AAG TT |

**Amplified condition for the *SLC14A1* by PCR-SBT**

The total PCR amplification volume is 20 µl reaction system, contains 1×LA PCR buffer, 0.2mM dNTPs, 1.6mM Mg^2+^, forward and reverse primers each 0.2µM, 1U LA-Taq (TaKaRa, Dalian, China), approximately 50ng DNA template, supplemented with H_2_O to 20µl. The PCR reaction procedure is 95℃ for 3 min, then 95℃ for 30 s, 61℃ for 30s, 72℃ for 70s, 35 cycles; finally 72℃ for 10 min.

**Supplemental Figure**

**Supplemental Figure 1 The full-length covering from 5' UTR to exon 10 in *SLC14A1* gene is amplified in two overlapping amplicons**

**Supplemental Figure 2 Jk^b^ antigen expression on the transfected cells using flow cytometry. Positive expression in the red box. NC: empty vector control; Jk^b^:c.838G>A vector; JK^V1^: JK(c.230G>A+c.499A>G+c.588A>G+c.838G>A) vector; JK^V2^: JK(c.499A>G+c.588A>G+ c.838G>A) vector.**
